# Improving Aboriginal Maternal Health by Strengthening Connection to Culture, Family and Community

**DOI:** 10.3390/ijerph17249461

**Published:** 2020-12-17

**Authors:** Paula Wyndow, Elaine Clifton, Roz Walker

**Affiliations:** 1 Research Consultant, Mount Claremont, Perth, WA 6010, Australia; wyndow@aapt.net.au; 2 Telethon Kids Institute, University of Western Australia, Perth Children’s Hospital, 15 Hospital Ave, Nedlands, Perth, WA 6009, Australia; elaine.clifton@telethonkids.org.au; 3 School of Population and Global Health, University of Western Australia, 6 Clifton St, Nedlands, Perth, WA 6009, Australia

**Keywords:** Aboriginal women, smoking cessation, pregnancy, trauma-informed, women-centred care, culturally meaningful, community based participatory action research

## Abstract

(1) Background: To explore the function of smoking in Aboriginal women’s lives from a trauma-informed, women-centred approach in order to inform the design of a culturally meaningful smoking cessation program for women living in the Pilbara, Western Australia; (2) Methods: Qualitative and Community Based Participatory Action Research (CBPAR) was used to discover what Aboriginal women know about smoking, the specific contextual issues that influence their smoking, and what community supports are available to help them quit smoking. Inductive analysis was used to determine key themes; (3) Results: 25 Aboriginal women (smokers, non-smokers, and ex-smokers) participated in focus groups or individual interviews. Women smoked to deal with stress, trauma and for maintaining social connections. Women who stopped smoking did so on their own when the reason was important enough or when they saw alternative ways of living. Creating safe places to bring women together to yarn about women’s business and link with health services was identified as critical to support women to stop smoking. Conclusions: Strategies to address smoking need to bring community, culture and health together in a meaningful way for women and their families; build on existing community strengths; and educate communities about the effects of smoking, and health professionals about how to support women to stop smoking.

## 1. Introduction

In 2013, 56% of Aboriginal women in the Pilbara, Western Australia (WA) reported smoking during pregnancy compared with 10% of non-Aboriginal women [1]. As well as the well-known adverse health effects of smoking on women’s health, smoking tobacco during pregnancy is associated with poor birth outcomes, including low birth weight, premature birth and perinatal death [2]. While there is recognition that Aboriginal women experience a range of adverse events including socioeconomic inequality and the ongoing traumatic effects of colonization, racism and the stolen generations [3,4], few studies have sought to address these factors when designing smoking cessation programs for young pregnant women and women of child bearing age. Moreover, few studies have considered the intersectionality of gender, trauma and culture in relation to Aboriginal women’s everyday lives [5]. Acknowledgement of the critical role of these complex interrelated factors in maintaining individual and family attitudes and behaviours towards smoking is what differentiates our project from others and is what makes it innovative.

As reported in an earlier paper, [5] the key aims of this project are to: (a) Explore the function of smoking in Aboriginal women’s lives, and specific contextual issues that influence their smoking such as trauma and violence, disadvantage, identity formation, social functions and stress; (b) Design and deliver a relevant, culturally meaningful smoking cessation program for women living in the Hedland and Western Desert communities in the Pilbara region of WA; (c) Address Aboriginal women’s smoking in an integrated and holistic manner, as part of the healthcare they receive before, during and after pregnancy, within a primary health care setting; and, (d) Enhance the evidence base on the effectiveness of a women-centred, trauma-informed approach to smoking cessation.

This paper reports on the findings for the first aim of the project, which was to explore the function of smoking in Aboriginal women’s lives and the specific contextual issues that influence their smoking, and then use this information to inform the design of a women-centred, trauma-informed culturally meaningful program of work to support women living in the Hedland and Western Desert communities in the Pilbara region of WA to reduce their smoking.

**Figure 1:**
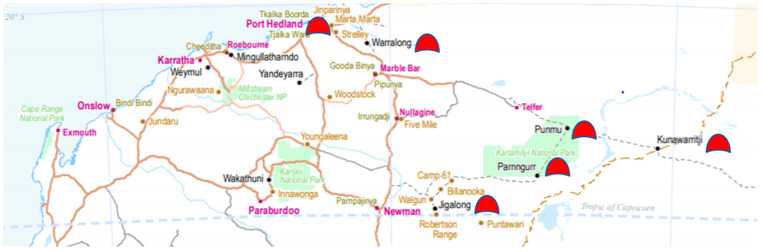
Aboriginal communities in the Pilbara involved in the project. Map adapted from the Department of Aboriginal Affairs 2019 Aboriginal Communities © DAA October 2019; https://www.dplh.wa.gov.au/DepartmentofPlanningLandsHeritage/media/Documents/Information_services/State%20Planning/Aboriginal%20Communities/ABL-Aboriginal-Communities-WA-map-Oct2019.pdf.

Facilitated through Rural Health West with funding from the Commonwealth Government, this research project, *Tackling Indigenous Smoking in the Pilbara*, was conducted as a partnership between academic researchers RW and PW who worked at the Telethon Kids Institute at the time, and tobacco prevention officers in Wirraka Maya Aboriginal Health Service in Hedland, and Puntukurna Aboriginal Medical Service in Newman, servicing four Aboriginal remote communities in the Pilbara region of WA. See Figure 1.

## 2. Materials and Methods

### 2.1. Participants and Methods

For the first stage of this study Community Based Participatory Action Research (CBPAR) was utilised to collect data and engage with Aboriginal women, health professionals, service providers and other stakeholders. As the purpose of this qualitative research was to inform the design and delivery of a trauma-informed program, it was important that the researchers sought to embody the principles of Trauma Informed Care and Practice (TICP) in the research process. These include: (1) understanding trauma and its impact; (2) creating a culture of safety and trustworthiness; (3) ensuring cultural competence; (4) supporting consumer control, choice and autonomy; (5) sharing power and governance; (6) integrating care; (7) understanding that healing happens in relationships; and, (8) believing that recovery from trauma is possible [6]. Utilising CBPAR enabled us to incorporate TICP principles throughout our research. CBPAR is a way of conducting research in which community members and academic researchers are equal partners in all stages of the research [7]. We were also committed to a decolonising approach aligned with the National Health and Medical Research Council conduct of research in Aboriginal contexts [8]. Central to this process was partnering with two Aboriginal Medical Services, Wirraka Maya Health Service Aboriginal Corporation (WMHSAC) in Hedland and Puntukurnu Aboriginal Medical Services (PAMS) in Newman and taking time to engage with the communities they service and the relevant community organisations and key stakeholders in those communities.

### 2.2. Data Collection and Community Engagement

Between February 2017 and November 2017, a series of small focus groups and individual interviews were conducted with Aboriginal women, health professionals and other relevant organisations in Hedland, Warralong, Newman, Jigalong, Punmu and Parngurr. Twenty-five Aboriginal women participated, including current smokers, ex-smokers, and women who had never smoked, as well as young women, young mothers, grandmothers and pregnant women. Whilst a range of methods were used to engage the community in the research project including community barbecues, films, use of posters with health messages, community workshops, and participation in maternal health and smoking prevention health promotion events, only three pregnant Aboriginal women were available to be interviewed, highlighting the significant challenge of engaging with this group of women. Whilst we experienced difficulty in employing an Aboriginal researcher for the entire study, in Hedland we consulted with the existing Aboriginal Community Reference Group (CRG), Aboriginal Health Workers and Tackling Indigenous Smoking (TIS) workers at WMHSAC and employed an Aboriginal researcher (EC) to assist with one of the focus groups with an Aboriginal organisation in Hedland. Two Aboriginal women assisted in recruiting women into the study in Newman through existing women’s wellbeing and healing groups. In addition, senior Aboriginal women leaders living in a remote community in the Western Desert attended a community meeting and encouraged women to participate in the study. We consulted with these women on a regular basis through all phases of the research.

Interviews with Aboriginal women were typically held at places that they were already attending or familiar with, including schools, Aboriginal Medical Centres, Early Years Centres, and Playgroups, with recruitment usually occurring through flyers distributed by service staff, advertisements through posters on community noticeboards or word of mouth by community members. A yarning approach was taken, firstly social yarning [9], then women were given verbal and written information about the research project, consent for their involvement was sought and then more formalised yarning was conducted.

The yarning sessions began with a general discussion about smoking. Participants were asked what they knew about smoking, what they liked and did not like about smoking, why they think people smoke, what they knew about quitting, and what they think is needed in their community to help people reduce their smoking and support women who want to quit, particularly in pregnancy. Participants were not asked directly whether they were current smokers as it was important that women participating were able to take the lead in what they wanted to share, rather than being placed in a position where they did not want to answer the question or felt uncomfortable in doing so. In most cases women revealed their smoking status further into the interview, as part of their response to questions around quitting or accessing support. In a small group, this information was sometimes offered by a friend or family member. Upon request from one of the communities, we presented a community workshop on smoking and provided feedback about what the women had told us about smoking in the interviews.

Over the same period key informant interviews and focus groups with health professionals and stakeholders were conducted to understand their perspectives of why Aboriginal women smoke and the barriers service providers face in providing effective support to women in the community who may need assistance to stop smoking. Twenty-five service providers took part in these conversations. Their responses will be reported in a future paper.

The two researchers (PW and RW) took turns to ask questions while the other scribed. As well as scribing individual responses, both researchers kept field notes to record observations and reflections made during interactions with women and other stakeholder in the communities. After each trip these were individually transcribed, and the data, observations, and reflections shared and validated through the data analysis process described below.

### 2.3. Data Analysis

Inductive thematic analysis was used to analyse the participant interview and focus group responses using a women-centred, trauma-informed lens. This general inductive analytic approach [10] established the links between the research objectives and the summary findings which emerged from participant responses. The adoption of a women-centred, trauma-informed lens revealed some of the key systemic issues and social determinants underlying the experiences and processes described by participants, which in turn facilitated a deeper understanding of factors that contribute to Aboriginal women’s behaviours and decisions related to smoking in pregnancy.

Themes were developed after reviewing participant responses from the initial consultations, and were then discussed with women on the next day or at the next visit. These themes were also raised and confirmed with each subsequent group. After all the consultations were completed a lay summary of themes was prepared for the participants, outlining what women told us about smoking which was shared with the women and health service providers and TIS offers. Additions were made throughout the iterative research process to ensure all key ideas and sub-themes were included, for example, issues related to what women thought would help them to quit smoking were continually added to reflect different perspectives.

These systematic procedures for analysing the data produced trustworthy, reliable and valid findings [10]. These themes were then reported back and confirmed by the groups in a number of settings including a community barbecue, three mums and bubs groups, and meetings with women leaders on Community Councils. In particular, EC had substantial input into the development and implementation of the pilot Weaving and Yarning program and was appointed as a Community Care Worker (CCW), both strategies which had evolved and been acted upon from the early findings of this CBPAR project. EC also contributed to and co-presented on the project at the Hot North Conference held in Hedland in June 2019 [11].

Drawing on reflections from our preliminary paper [5] participant interviews, a review of the trauma-informed, women-centred literature, and local issues and perspectives arising at the Hedland workshops conducted by international experts Nancy Poole and Lorraine Greaves (in partnership with another Telethon Kids Institute study, *Making FASD History in the Pilbara*), we developed future recommendations that acknowledge the intersection of trauma, gender and culturally informed principles and practices [5,11,12].

As part of the CBPAR process, two workshops for health professionals and Aboriginal community members were conducted with information on women-centred and trauma-informed approaches to smoking and maternity care and practice. Eighteen men and women attended the community workshop and five WMHSAC staff. Elders and community members attended a second workshop.

### 2.4. Ethics

This research study received ethics approval from the Western Australian Aboriginal Health Ethics Committee—Project Reference 744.

## 3. Results

An analysis of the transcripts from the consultations with community participants revealed the following key themes and sub-themes: the first theme related to the function of smoking in women’s lives including the normalisation of smoking, experiences of stress and trauma in their lives, boredom, and Lack of Purposeful Activity and the link between smoking and drinking alcohol. The second theme related to stories about quitting and the third theme centred around what women wanted to be able to quit smoking.

### 3.1. The Function of Smoking in Women’s Lives

Women talked about why they first took up smoking, why they still smoke and, for those who had successfully quit, the reasons they ‘gave up the smokes’. Many women started smoking at a young age (12–15 years) as a result of peer pressure and because other family members smoked.

#### 3.1.1. Normalisation

The normalisation of smoking in people’s lives was a recurring theme.

*Key thing about smoking is the normalisation of it for children growing up—if everyone smokes around you then that’s your norm.*

P009

*Everybody smokes, kids start smoking really young with their brothers and sisters, at about six some start, then they get addicted can’t give it up when they want to play football or have a baby, young girls have babies young around fifteen, maybe eighteen.*

P014

One woman said that until they went to school they did not know about the negative health effects of smoking, nor did they realise that smoking marijuana was not a normal thing to do, or even that it was illegal. P003

#### 3.1.2. Stress and Trauma

The presence of stress and experience of trauma in their lives was a key factor that influenced women’s smoking. For some women, going outside for a smoke was, ironically, the one thing that gave them a sense of control over their lives. Older women talked about using smoking to calm themselves when things got hard.

*People smoke more when stressed*,

P006

*The women and girls here have a lot of stressors. We lost one of our young men a few weeks ago he was killed in a car accident on the way back from town. Everybody gets sad, there is a lot of grief for mothers and for grandmothers and young girls too, they miss him. We need to have somewhere safe here where women can go, where they can come together and yarn and share stories and help each other.*

P024

#### 3.1.3. Boredom and Lack of Purposeful Activity

Younger women reported boredom as a key factor in continuing to smoke and spoke about how there used to be a lot more programs and fun activities for them. The lack of programs and activities, particularly in smaller communities, was raised repeatedly. In relation to programs, services, activities and resources, women often said “*We used to*…”

*We used to play lots of music, drums, ukulele, singing, performance. Dance hip hop.*

P015

*We used to do cooking, dying our hair, camps.*

P015

*We used to go out on country, on Fridays we would collect traditional bush medicine.*

P016

*We used to have our own bakery and markets on Saturday morning.*

P017

*We used to take the girls out to a special place, talk to them about culture and family.*

P022

Young women fulfilling family obligations, caring for their younger siblings, were generally not engaged in any work outside the home or in educational activities. One community member made a number of observations to address such issues in her town and to create opportunities and strengthen hope and aspirations.

*It’s important to keep the Aboriginal playgroup going now that [stakeholder] is leaving. …Young girls need to be in a routine every day… need to take young women out to other communities to see what’s working well.*

P012

Similar, concerns about the need to have playgroups and days care to give young mums some respite, and maybe links with agencies, were raised by some women in Hedland.

#### 3.1.4. Link with Alcohol and Socialising

When talking about smoking women also talked about drinking alcohol. One woman reported that she would go and buy two packets of cigarettes before going out drinking because she knew she would want to smoke. P008. Another woman said she drank to:

*Keep that bad feeling away”, but at the same time drinking made things worse as sometimes it would bring about that bad feeling, and then I would feel bad about drinking.*

P007

#### 3.1.5. Link with Yarning and Stories

The main benefit of smoking for women was that it was linked to yarning and telling stories and that this was important in relieving stress and connecting to a sense of community. In town drinking alcohol and smoking were associated with having a good time—having a laugh, again related to relieving stress and worries. However, women also acknowledged the downside, including the cost and feeling bad sometimes.

### 3.2. Stories About Quitting

Stories about quitting related to the role of stress as a barrier and a side effect of quitting, not knowing how to quit, and the difficulties of quitting when other people around them smoked. Going cold turkey seemed to be the main way people knew how to quit.

#### 3.2.1. Feeling Stressed and Cranky from Withdrawal

Most women who were smoking at the time of the interviews said that they had made attempts to slow down or quit smoking but found it difficult, particularly when they were feeling stressed, anxious, upset and worried. Three young women in one of the Western Desert communities said that most women smoke to stay calm and to share stories and be social. They agreed that giving up is really hard. One young woman who was pregnant talked about trying to give it up.

*It’s really hard, the baby Doctor, when she came here last time she told me I have to give up for the baby, I feel really bad, yeah I feel it and shame and all and worry all the time. I don’t want my baby to be born premmy, or have weak lungs. I am slowing down and I want to give up smoking but it’s really hard, it makes me stressed when I try to stop.*

P024

Withdrawal symptoms from quitting attempts also made people cranky and bad tempered and women worried about other people’s ability to manage their emotions and behaviour when they weren’t smoking, whether it was because they had run out of smokes before pay day or were trying to give up. Several women said they see that, all the time, people stress without smokes,

*You see women yelling at their kids when they run out of smokes.*

P004

*Women smoke because of stress. They get into arguments then have a smoke to calm down, I don’t think people realise what happens to them when they stop smoking—the withdrawal.*

P014

Several participants spoke about the difficulties of stopping smoking when people around them still smoked, especially their partners.

*He just has them on the table.*

P002

*It’s hard when people smoke around you, My partner, he tried to quit, he gets cranky. He need that smoke.*

P005

#### 3.2.2. Not Sure How to Quit

Women were asked about the support they had received in stopping smoking. Most women reported that they did not really know how to smoke less or quit. A couple of women had tried nicotine patches but found them too sweaty; another woman said she had used a quit spray but that it did not work. Another woman said that the quit spray had worked for her but not for her partner. Whilst participants had heard of nicotine gum they had not seen or used it. Seeking help from the TIS officer/s or the Aboriginal Quitline was not reported. Some women had talked to their doctor or midwife but had been reluctant to go to the TIS officers for counselling.

#### 3.2.3. Going Cold Turkey

Quite a few women had successfully quit smoking, and most of them had quit cold turkey with no formal assistance. It appeared that women were able to quit smoking when the reason for giving up was big enough. For example, one woman quit smoking when she was diagnosed with cancer, and another woman quit when her baby was born premature and “sickly”. Several of the mums with young children said that they had quit smoking because they wanted to be good role models for their own children.

*I don’t want my children to even know what smoking is I tell my family to smoke outside, I don’t want my daughter to even look at them smoking…. My little daughter tells them “don’t smoke in her house”.*

P001

Several women said that they made positive changes to their lives when they went to stay with other family members and saw a different way of living. This was reiterated by one of the older women who felt it was important to take young girls out to other communities to see what was working well and to see other ways of living. Reflecting on her smoking journey during the interview, one woman stated that it was her involvement in sport and having one person who really believed in her that was the catalyst for her making more positive choices in her life. Another woman had slowed down her smoking during pregnancy and was planning to quit because she had a career goal she was working toward.

*I want to quit because I have a lot of things on my mind.*

P007

Several women spoke about the positive things their partners and family members were doing to make their lives more smoke free and to reduce their family’s exposure to second and third hand smoke, for example, smoking outside, not smoking in the house or car. One woman recounted how when she takes her baby to visit her Nan (who has smoked since she was 12-13 years old), her Nan puts on fresh clothes, sprays herself, washes her hands and puts on cream to get rid of the smell of smoke.

One woman thought that other women eventually stopped smoking and drinking because,

*they have had enough of it, don’t’ wanna be like that, [they have] seen too much of it.*

P004

### 3.3. What Women Want

Women were very clear about what would help them stop smoking. This included knowing the facts about smoking in a culturally appropriate way, “*not making you feel more scared and no good*”; and health promotion to help prevent young people from starting smoking. Connecting to culture and recognition of diversity were also identified as important, along with having a safe place to go.

#### 3.3.1. Women Want to Know the Facts about Smoking in a Culturally Relevant Way

Several women expressed concerns about the negative health impacts of smoking on their lives. They raised concerns about it affecting their breathing, “*getting puffed out*”, or “*catching cancer*”. These concerns were in relation to their own smoking, but also from other people smoking around them—second-hand smoke. Women said that they would like more information about the facts associated with smoking, its direct health effects and its relationship to other diseases, e.g., diabetes. Participants also stressed the importance of having clear and consistent messages about smoking and its effect on health for both mothers and their babies, “but not in a way that judges or shames people”. More education was needed in the community about smoking and drinking, but in ways that have meaning for people.

One teenager (17 years), who was pregnant, had never smoked and was clear that she would never take it up, especially now she was having a baby, as she wanted a healthy baby. Yarning with her she suggested that,

*It’s really good to have these community days with Wirraka Maya, involving the school and everyone and the old people, having a BBQ with roo tails and special fun games to show young kids and parents the dangers of smoking. These things will maybe stop the young kids from six up to ten or eleven from smoking. It’s good for the kids at school to learn their rights too you know to have smoke free homes so they don’t get sick or start smoking—they can take their photos home* (pointing to the smoke free home photo booth with the kids lined up) *and put them up on the fridge to show people who comes to their house not to smoke…*

*Yeah I won’t smoke ever or let people smoke around me for my baby.*

P024

This young woman was referring to some of the games and activities being conducted by the Wirraka Maya Social and Emotional Wellbeing team and TIS workers at a community smoking awareness event on World No Tobacco Day. One of the most popular activities for kids was having their photo taken in the ‘smoke free home’; there were also many activities conducted throughout the day that involved culturally safe messages promoting the links between smoke free living with family and community health and wellbeing, which kept the community engaged.

#### 3.3.2. Women Want Action to Prevent Young People from Taking Up Smoking

Older women spoke about their regret at taking up smoking and that this was something they did not want for their children and grandchildren. They spoke about the importance of preventing young people from starting smoking in the first place, so they did not become addicted to cigarettes. They requested more education about smoking in schools from a young age, e.g., 6–7 years, regular smoke free events with information about smoking, and the creation of more smoke free places in the community.

#### 3.3.3. Women Want Greater Connection to Culture

Many women spoke about the benefits of spending time on country and the importance of taking young people out there regularly. Young women spoke passionately about going on country to hunt and forage for traditional bush tucker—e.g., bush potatoes, Wichita grubs, honey ants. This was something that held happy memories for them and something that they knew a lot about.

“*It feels better to be on country—feels more better in your body*”

P007

Women in the Western Desert communities worry that because of the drinking and smoking, Martu culture is slowly getting lost. Younger women want stronger connections to Elders in their community. They spoke of wanting to hear Elder’s stories, to find out what the Elders were doing when they were younger. The women also wanted to learn more about the bush and the country and bush medicine. Others spoke of the need for stories and health information written in Martu.

*We need books and information about the effects of smoking and drinking for Martu written in Martu.*

P022

Women also wanted activities that were culturally meaningful. Several women expressed concerns that there was very little purposeful activity for them in the communities. Some suggestions were for women to take their children out bush and teach them about culture, bush tucker, traditional stories and the water holes. One grandmother said all pregnant young girls should go out with Elders and the women rangers so they could teach them about having healthy babies.

*Need to have KJ rangers take 10 to 20 teenagers out bush and get healthy food turkey, goanna, kangaroos, honey ants.*

P022

The Arts, painting, dancing, music, crafts, were also identified as important ways for people to heal by strengthening connection to culture.

#### 3.3.4. Recognition of Diversity Amongst Aboriginal People

A couple of women who were working hard to “*grow their children up healthy and strong*”, expressed their concern about their children being exposed to negative stereotypes of Aboriginal people in the media, especially social media trolls, and people’s assumption that all Aboriginal people smoke, drink, fight and can’t hold down jobs.

*It is the negative stereotypes that perpetuate people feeling bad about themselves and feeling low and that contributes to the racism.*

P001

They want Aboriginal young people to recognise and understand that all Aboriginal people are not the same and that they do not have to act the way others expect them to. In trying to break the cycle in her family one young woman talked about,

*the importance of there being more than one story—that there were many stories…we can make our own story.*

P001

#### 3.3.5. Safe Places for Women’s Business

Overwhelmingly, women spoke about having a women’s place, a safe space where they can gather to yarn about their worries and their stressors and engage in meaningful activity. Women spoke of the need for a women’s centre or space in which to conduct women’s business, to be busy and feel supported by other women. Several communities had previously had women’s centres, which, for a range of reasons were then closed.

Some of the activities women said that they would like to be involved in included arts and crafts, weaving, sewing, cooking, dancing, making films, music, and performance opportunities and going on camps. There was general agreement that while women were engaging in activities, they would be less likely to smoke, drink or take drugs.

*Women smoke less when painting, smoke worse when drinking.*

P006

Several women were keen to get a group together to support young women when they first set up house. They recognised that some young women would need some help with setting this up, however, they were determined to drive this and help women to feel proud and in control.

*They need a plan. They need help to learn how to look after their kids and home; they need to learn how to refuse entry to people going there, they need support with the cleaning.*

P012

## 4. Discussions

Adopting a women-centred, trauma-informed approach to support Aboriginal women in the Pilbara to identify the best ways to slow down or stop smoking in pregnancy highlighted the importance of developing a program of work focusing on key processes and elements to address the social determinants which serve as both risk and protective factors influencing women’s health seeking behaviours. Consistent with earlier research, the normalisation of smoking in Aboriginal women’s lives and its value as a tool to manage stress and trauma and to promote and maintain social connection and relationships with friends and family [13,14,15,16,17] suggests that for many women the perceived benefits of smoking outweigh any adverse health outcomes for themselves or their family. Similar to other studies [18], it seemed evident from the discussions with women and health professionals that many women experienced depression and anxiety, which made it difficult to quit smoking. The work of Lorraine Greaves, a medical sociologist involved in research exploring the intersection of trauma and gender through the Centre of Excellence for Women’s Health in Canada, supports this finding. Her research revealed the many ways women construct meaning around smoking in their lives, including organising social relationships, creating an image, controlling emotions, exercising dependency and creating identity [19]. Women in our study, as with other studies [17], primarily used smoking as a coping strategy to manage emotions and maintain relationships, and to deal with stress, worry and complex trauma. For younger women not engaged at school or work, or caring for younger siblings, smoking relieved the boredom and gave them something to do, including socialising. Furthermore, as a national TIS program evaluation notes, “For communities facing a range of complex issues which demand attention, for example, family and domestic violence, trauma and depression, and drug and alcohol misuse, prioritising smoking can be a significant challenge.” [16] (p. 82).

To date, culturally responsive, women-centered approaches to smoking cessation have shown the most promise. These are comprehensive, holistic approaches that recognize gender as a social key determinant of health [20]. Mainstream smoking cessation strategies are often targeted at the individual; however, our findings show that for Aboriginal women smoking is a social activity. The role of women’s social environment, especially partners as key influencers of smoking behaviour, both positive and negative, has been previously reported [13,15], with a recent NSW study finding that mothers are also key influencers [18].

Aboriginal women’s relationships are important for their social and emotional wellbeing [14], therefore smoking cessation strategies that are inclusive of other family members need to be considered as part of any smoking cessation strategy. Gender transformative approaches to smoking cessation encourage uptake of smoking cessation interventions along with a wider range of non-stereotypical gendered roles that include fathering for men and work for women as potential motivators. A study by Gould et al. [21] also confirmed the importance of family and community level interventions, thus making smoking everyone’s business.

### 4.1. Many Women Are Taking Positive Steps Towards Smoking Cessation

One of the central tenets underlying a trauma-informed approach is that it is strengths-based rather than deficit-based. From the consultations it was really important to acknowledge that many women were already taking positive steps towards creating a more smoke free life for themselves and their families. This included making their homes smoke free and encouraging family and friends not to smoke around their children. Some women had already quit smoking, some for a couple of years. This response is similar to other studies with Aboriginal women, including a systematic review related to smoking cessation, that identified that women often assume/create a sense of agency to give up smoking when they find out they are pregnant in order to achieve well-being, give their baby the best start, and to take pride in being a healthy mum and female role model [17]. Young mothers were focused on being strong role models for their children, teaching them about meditation and being healthy and strong, reinforcing other research that found that for some young Aboriginal women pregnancy has the potential to be transformative [18,22,23]. Having someone who believed in them and seeing other healthier ways to live were identified as critical factors in successfully stopping smoking. Acknowledging and validating the positive actions that women are taking, no matter how small, is important in building self-efficacy and confidence. Being supported to make these changes then allows women to start to change their smoking story.

### 4.2. Smoking Needs to be Addressed as Part of Holistic Antenatal Care in Pregnancy

Another important finding was the need to address smoking in a holistic way as part of a whole package of antenatal care in pregnancy. For example, people who smoke cigarettes are also more likely to drink alcohol and use drugs [24,25]. Several women in this study reported smoking more when they were drinking alcohol, thus addressing alcohol consumption in pregnancy as part of preventing its harmful effects on the mother and baby may be a potential strategy to reduce smoking rates too. Early smoking initiation is linked to a higher risk of multi-drug use in pregnancy [25], thus focusing on preventing women from taking up smoking in the first place is critical. Building positive stories of young women making good choices was a focus of two programs being offered through both AMSs at this time. Funding was obtained to pilot a Weaving and Yarning program for bringing pregnant women, their mums, grandmothers and aunties together with Elders to yarn about healthy mums and healthy bubs, as well as providing a culturally secure link to health providers.

### 4.3. Creating Safe Women’s Places

The reopening and re-imagining of safe women’s places, both in communities and in towns, to engage in meaningful work and to yarn about their worries, support each other and share stories was called for repeatedly during the consultations. As with other studies [17], the results of this study confirm that for many women the ongoing impact of stress, worry and anxiety, grief and trauma makes it difficult to quit smoking and to provide self-care [18]. The need for a safe place that women can go to if they are at risk of being harmed or harming their baby by drinking was identified as critical by women. Although not the focus of this paper, this point was also reinforced by health professionals and other service providers working closely with women. In particular, many of the discussions with women and health professionals confirmed the need for gender and trauma-informed services and prevention strategies. These included the need for women to have access to safe places such as Women’s shelters and women-only hostel accommodation when travelling to Hedland to have their baby, to remove themselves from family and domestic violence and family pressures, or in some cases to address harmful alcohol consumption identified by midwives using the Alcohol Use Disorders Identification Test (AUDIT-C), an alcohol screening tool in antenatal settings. The idea of providing a safe place for women to go to protect themselves and their unborn child/ren, rather than supporting them after they have been victims of, or witnesses to violence and abuse, is critical to empower and support women to take preventative action.

### 4.4. Many Women Quit on Their Own

Importantly, the study showed that many women were able to quit when the reason was important enough and most women who quit stopped smoking on their own, without any professional help. It is unclear whether this was because women were unaware of existing smoking cessation strategies such as counselling or Nicotine Replacement Therapy (NRT), with providers preferring to suggest more holistic approaches that acknowledge the underlying reasons for women’s smoking. National and international guidelines for smoking cessation in pregnancy recommend behavioural counselling, followed by NRT for women who need more support [26]. However, few women in our study had used NRT and those that did reported little success with it. In one site the NRT referral process was complex and women were required to have several appointments with several people including the GP, the midwife or nurse practitioner and the TIS counsellor, and some were referred to drug and alcohol services.

### 4.5. Many Women Were Unaware of Support Available to Them

It seems that, overall, women were unaware of the specific help available to them, or had little interest in accessing direct assistance, i.e., counselling to quit smoking. This was also evidenced by very few people accessing Quitline. This may be because women fear being judged for their smoking as suggested in other relevant studies [17,27,28], do not have access to credit on their phones, or are simply unaware of the service. The online video by the Quitline Aboriginal Liaison Team (QALT), located at the Aboriginal Health Council of Western Australia (AHCWA) may be a useful tool for health professionals as part of their Brief Intervention to show women what they can expect when they call the Quitline. It is also an opportunity to show how the Aboriginal counsellor on the other end of the line works in a gentle, compassionate and culturally secure way with callers, using narrative therapy to support people who are interested in quitting.

As Bovill (2020) writes ‘Aboriginal and Torres Strait Islander women *are quitting smoking during pregnancy and care deeply about the health and wellbeing of their babies*’ [29] (p. 358, emphasis added). Women want to know the facts about smoking [17]. Consequently, providing the whole community with the facts about smoking through community barbeques and local video messaging, makes smoking “everyone’s business”, not just the responsibility of the individual.

There are so many complex factors that sustain women’s smoking; quitting, i.e., going cold turkey, can seem overwhelming and numerous unsuccessful attempts can be demoralising. A recent study found that for heavy smokers it takes on average 17 quit attempts before a person succeeds in quitting [30]. Therefore, ensuring that women are aware of the wide range of positive behaviours they can do while trying to quit is really important in shifting their mindsets and those of health practitioners from one of “either success or failure”, to one that acknowledges the lived experience of women’s lives and bears witness to both their successes and setbacks on the way to quitting. Through our consultations we identified over 40 different possible activities women could do to help quit smoking. These included: building women’s health literacy about the specific health effects of smoking, including second and third hand smoke; improving their help-seeking behaviours—i.e., watching YouTube videos about the Aboriginal Quitline and making a call to them; practising talking to family and health providers about their smoking journey and asking for help; practising self-care, e.g., creating some smoke free time each day; asking family not to smoke around them, eating good tucker; practising community care, e.g., encouraging a family member or friend who is trying to quit; creating a smoke free home and car; aspiring/dreaming about a healthier future, e.g., making achievable goals, rewarding themselves, learning a new skill; identifying and implementing quitting strategies, e.g., making a quit attempt, downloading quit apps, trying nicotine gum or patches, and slowing down their smoking.

Despite mass media campaigns about the risks of smoking, women’s comments reflect the importance of not assuming that everyone knows or understands the links between smoking and adverse health outcomes. Importantly, connecting with culture, going on country, and yarning, weaving or painting were frequently raised by women as strategies to promote healthy pregnancy, and to reduce smoking and drinking. Several women expressed interest in using locally produced videos that focus on healthy futures for mums and bubs; they also loved seeing the Puyu Paki video produced by Jigalong community.

The work of Nancy Poole and Lorraine Greaves supports our findings. They demonstrated that to be successful and effectively engage women with complex care needs that smoking cessation strategies/interventions need to be trauma-informed and designed to address multi-level factors simultaneously [31]. They propose four tailored approaches, woman-centered, trauma-informed, harm-reducing, and equitable, in order to address smoking in young and socially disadvantaged women spanning pre- and post-pregnancy periods, as well as acknowledging women’s social contexts and concerns.

The *Bringing them Home* report twenty years on calls for the continuance and expansion of healing approaches to reduce the negative effects or symptoms caused by intergenerational and historical trauma [32]. The expansion of Healing circles across the country and the blending of Indigenous healing and western healing approaches show some promise in reducing severity of symptoms, and recommend locally based healing solutions developed by mainstream and Indigenous healers working together [32,33,34]. Such strategies are crucial to support a holistic approach to smoking cessation.

The trauma-informed workshops held in Hedland provided TIS and other health practitioners with strategies and resources for assisting women, particularly pregnant women, to reduce or quit smoking. At the end of the consultation process a Weaving and Yarning program was established in partnership with the Spinifex Hill Studio, Child and Parent Centre and WMHSAC in Hedland and discussions were well underway with relevant groups in Newman and Jigalong.

### 4.6. Limitations

There were several limitations to this study, the first one being the difficulty in engaging with, and having access to, Aboriginal pregnant women in Hedland, Newman and the Western Desert. This was an ongoing issue throughout the study despite a range of strategies to try to address this, including going to places where women were accessing services, consulting with Elders and the CRG, and regularly meeting with key project partners and the health professionals engaged in their antenatal care. One of the biggest challenges was the high turnover of staff, particularly in Hedland, and the expressed concerns of some staff that asking women to talk about smoking and drinking in pregnancy may create additional stress and stop them attending the clinic, and women often not presenting for care until later in pregnancy.

## 5. Conclusions

One of the key strengths of this study was the research approach taken. The critical value in using this approach meant that the authors were able to raise issues identified by participants with the relevant stakeholders to facilitate positive changes throughout the project. For instance, taking a women-centred, trauma-informed approach to smoking cessation revealed that women want safe places in their communities where they can engage in meaningful activities and yarn with other women. Over the course of this CBPAR study the focus on these issues resulted in one of the communities re-establishing a women’s centre and others started working towards securing women’s places to provide a safe space and important opportunity for health providers and other agencies to work with women to address some of the underlying social determinants of health. In another instance, a women’s shelter in Newman reviewed their practice, incorporating therapeutic strategies to promote healthy choices around reducing substance use, and encouraging women to attend the shelter as a prevention to domestic violence and trauma.

The trauma-informed workshops held in Hedland provided TIS and other health practitioners with strategies and resources for assisting women, particularly pregnant women, to reduce or quit smoking. At the end of the consultation process a weaving and yarning workshop was established in partnership with the local art studio and WMHSAC in Hedland and discussions were well underway with relevant groups in Newman and Jigalong. At the request of women in the Western Desert we arranged to show a locally produced video (Healthy Lady, Health Baby) to women in two of the communities, which proved to be very successful, with women asking that additional copies be made available to PAMS, the Ashburton Women’s Centre and World Vision mums and bubs group in Jigalong to support health promotion around smoking cessation and having healthy pregnancies. Similar to studies elsewhere in Australia, the above examples affirm the importance of developing support strategies that bring together both ‘traditional and contemporary knowledges, values and practices’ [29] (p. 359). A resounding theme in discussions with women was the need for holistic smoking cessation programs with a focus on strengthening connection to family, community culture and Country and promoting social and emotional wellbeing.

Some learnings from conducting trauma-informed research include the importance of approaching people with compassion, and recognising that women and their families in remote communities live in dynamic, often challenging environments, with many carrying excessive physical burdens as well as experiencing suboptimal social and emotional wellbeing. Many people in these communities have been “researched” many times and there is a perception that nothing ever changes. To address these concerns there is a need for researchers to reinforce the importance of restoring and maintaining relationships, practising deep listening, staying in the present, and not making assumptions about what Aboriginal women know. It also requires acknowledging and building on the positives and strengths in communities; practising critical reflection, reviewing each exchange or event and asking how it could be done better in the future. Meeting women where they are at is important, yarning informally about how they are going and acknowledging their needs and aspirations before starting a more formal research yarn [9].

Reducing smoking in pregnancy among Aboriginal women is a national priority in Australia [16]. Developing a smoking cessation program for Aboriginal women in the Pilbara requires a comprehensive program of work where the intersection of culturally responsive, gender and trauma-informed approaches, principles and practices are integrated to inform the multilevel strategies and initiatives that health professionals, services and program providers and policy and funding bodies need to implement for more effective women’s smoking cessation.
